# Core/Shell Conjugated Polymer/Quantum Dot Composite Nanofibers through Orthogonal Non-Covalent Interactions

**DOI:** 10.3390/polym8120408

**Published:** 2016-11-24

**Authors:** Brad W. Watson, Lingyao Meng, Chris Fetrow, Yang Qin

**Affiliations:** Department of Chemistry and Chemical Biology, University of New Mexico, MSC03-2060, 1 UNM, Albuquerque, NM 87131, USA; watsonbr@unm.edu (B.W.W.II); lmeng@unm.edu (L.M.); mefetrow@q.com (C.F.)

**Keywords:** organic photovoltaics, conjugated polymer, self-assembly, quantum dot, nanostructure

## Abstract

Nanostructuring organic polymers and organic/inorganic hybrid materials and controlling blend morphologies at the molecular level are the prerequisites for modern electronic devices including biological sensors, light emitting diodes, memory devices and solar cells. To achieve all-around high performance, multiple organic and inorganic entities, each designed for specific functions, are commonly incorporated into a single device. Accurate arrangement of these components is a crucial goal in order to achieve the overall synergistic effects. We describe here a facile methodology of nanostructuring conjugated polymers and inorganic quantum dots into well-ordered core/shell composite nanofibers through cooperation of several orthogonal non-covalent interactions including conjugated polymer crystallization, block copolymer self-assembly and coordination interactions. Our methods provide precise control on the spatial arrangements among the various building blocks that are otherwise incompatible with one another, and should find applications in modern organic electronic devices such as solar cells.

## 1. Introduction

Over the past few decades the application of conjugated polymers in organic optoelectronic devices, including organic photovoltaics (OPVs), organic field-effect transistors (OFETs) and organic light emitting devices (OLEDs), has received intense research efforts [1]. A significant portion of the research has been directed toward OPVs that have been considered as an inexpensive and flexible platform to supplement and even replace the traditional inorganic photovoltaic modules [2,3]. The fruits of such efforts have been OPV devices yielding record-breaking power conversion efficiencies (*PCE*) of over 10% [4,5,6,7,8,9,10]. Despite the initial lab-scale success, widespread implementations of OPV devices have been hampered, mostly due to the still relatively low *PCE*s, as well as non-satisfactory device stability, when compared to those of their inorganic counterparts.

Typically, OPV devices adopt the multi-layer structure, among which the light absorbing active layer is composed of two materials: an electron donor (conjugated polymers and/or small molecules) and an electron acceptor (typically fullerene derivatives). Active layer morphologies of the blends of electron donors and acceptors play decisive roles on device performances. In the current state-of-the-art single junction OPVs, electron donors and acceptors are simply mixed to form the so-called bulk heterojunction (BHJ) morphologies having interpenetrating networks with domain sizes on the order of 10–20 nm [11,12,13]. An ideal BHJ morphology maximizes the donor/acceptor interfacial areas and minimizes the distances through which excitons have to travel to reach an interface, while providing un-interrupted conduction pathways for both electron and holes towards corresponding electrodes. However, since it is a simple mixing of the active layer’s components, precise control over the morphology is difficult to achieve. Traditionally, solvent and thermal annealing processes have been applied to gain control over the active layer morphologies. These methods are time consuming and use trial and error approaches to find the optimal conditions for each combination of donor and acceptor materials, which may vary from system to system and lab to lab, and thus poses difficulties in repeatability and mass production. Furthermore, the optimized donor/acceptor mixtures are at best in a thermodynamically meta-stable state that deteriorates over time and reduces the device long-term stability.

There have been significant research efforts devoted to developing novel methodologies for constructing controlled and stable BHJ morphologies. One of the most studied methodologies toward stable BHJs in polymer solar cells (PSCs) is self-assembly of conjugated block copolymers (BCPs) having electron acceptors selectively attached to one block [14,15,16,17,18,19]. Most existing examples of this type have fullerene derivatives attached to conjugated backbones via covalent linkages [20,21,22,23,24]. Fullerene concentrations in these examples are generally low due to limited solubility and strong aggregation tendency of fullerenes. An intriguing alternative approach is to attach fullerene acceptors onto conjugated polymer backbones non-covalently, by which fullerene loading percentages can be easily adjusted and solubility of the resulting complexes can be enhanced. Several recent reports have described complexation between fullerene derivatives and polythiophene based diblock and random copolymers through hydrogen bonding [25,26,27,28] and π–π [29] interactions. We have recently developed a facile methodology that precisely nanostructures polythiophene fullerene molecules into well-ordered core/shell composite nanofibers through cooperation of several orthogonal non-covalent interactions including polymer crystallization, BCP self-assembly and complementary hydrogen bonding interactions [27,28,30,31,32]. Such composite nanofibers not only display controllability on BHJ morphologies at both the microscopic and macroscopic scales, but also lead to thermally robust devices. However, the device efficiencies are intrinsically limited by the narrow range of visible photons that can be absorbed by the poly(3-hexylthiopphene) (P3HT) polymers employed. In order to further increase the light absorption windows, additional light absorbers that are complementary to P3HT are needed.

Inorganic quantum dots (QDs) have recently become an extremely active research area owing to their unique electronic properties resulting from size-dependent quantum confinement. Tunable bandgaps through size variation, multiple exciton generation (MEG) processes, as well as large dielectric constants are all considered beneficial properties for enhancing third-generation solar cell performances [33,34,35,36]. Extensive research efforts have thus been devoted to QD solar cells, especially organic/QD hybrid solar cells employing QDs as either electron donors or acceptors [37,38,39,40,41,42,43]. However, respective electron and hole transport through these QDs is usually problematic due to the commonly present capping ligands and dis-connectivity between discrete QDs and aggregates. Furthermore, most of these hybrid devices are fabricated by simply blending the organic/inorganic materials, affording little control over the active layer morphology. The strong aggregation tendencies of QDs usually lead to macro-phase separation within the blend films and cause device deterioration over time. We envision that, by using our composite nanofiber (NF) strategy, we can incorporate QDs into the polymer/fullerene core/shell structures through non-covalent interactions to form ternary composite NFs. By carefully selecting QDs possessing band edges situated respectively between the lowest unoccupied molecular orbital (LUMO) and highest occupied molecular orbital (HOMO) levels of P3HT and fullerenes, we can decouple the QDs from charge transport and make them solely the complementary light absorber.

For a proof of concept, we report in the paper the synthesis and characterization of a new BCP containing a P3HT segment and a polythiophene segment functionalized with pyridine moieties at the termini of side-chains (BCP3). Self-assembly of BCP3 was achieved using a mixed solvent approach and the attachment of CdSe QDs with different capping ligands to form the core/shell organic/inorganic hybrid NFs through coordination and H-bonding interactions was demonstrated.

## 2. Experimental Section

### 2.1. Materials and General Methods

All reagents and solvents were used as received from Sigma Aldrich (St. Louis, MO, USA), Alfa Aesar (Tewksbury, MA, USA) or TCI America (Tokyo, Japan) unless otherwise noted. Anhydrous tetrahydrofuran (THF) was distilled over sodium using benzophenone as an indicator and was collected in flame-dried, air-free storage flasks. All NMR spectra were recorded on a Bruker Avance III 300 MHz spectrometer (Billerica, MA, USA) and referenced internally to the residual solvent signals. Size exclusion chromatography (SEC) was performed on a Waters 1515 system (Milford, MA, USA) equipped with a 2414 refractive index detector and a 2707 auto-sampler. The mobile phase was chloroform with 0.5% (*v*/*v*) triethylamine passing through two styragel columns (Polymer Laboratories, 5 μm Mix-C) at a flow of 1 mL/min, kept in a column heater at 35 °C. SEC results were calibrated by external polystyrene standards (Varian). Ultraviolet-visible (UV-vis) absorption spectra were recorded on a Shimadzu UV-2401 PX spectrometer (Kyoto, Japan) over a range of 300–900 nm using quartz cuvettes. Infrared spectra were generated by a Bruker Alpha-P spectrometer (Billerica, MA, USA), using a powder sample in ATR mode. TEM images were produced by using a JEOL 2010 microscope (Peabody, MA, USA) with a lanthanum hexaboride beam source and Gatan camera while in bright field mode. Samples were prepared by drop casting diluted sample solutions onto a carbon coated copper grids.

### 2.2. OPV(Organic Photovoltaics) Fabrication and Testing

Blend solutions were prepared by dissolving predetermined weight ratios of polymers, quantum dots and phenyl-C_61_-butyric acid methyl ester (PCBM) in chlorobenzene and the solutions were heated for 1 h at 90 °C in a nitrogen glovebox. The solution was then taken off the heat and stirred for 1 h, when a predetermined amount of “bad” solvent, i.e., acetone was added dropwise via a micropipette. This solution was stirred at 400 RPM for 9 h. ITO-coated glass substrates (China Shenzhen Southern Glass Display Ltd., Shenzhen, China, 8 Ω/☐) were cleaned by the following procedures: 15 min ultrasonic treatment sequentially in detergent, DI water, acetone and isopropyl alcohol and then UV Ozone (Novascan PSD series) treatment for 45 min. The substrates were then transferred to a nitrogen glovebox and 10 nm of MoO_3_ was deposited using an Angstrom Engineering Amond deposition system with a vacuum level of <7 × 10^−8^ Torr. The blend solutions were then spun cast on to the MoO_3_ surface at 500 RPM for 30 s. Aluminum electrodes were then added to the device via thermal evaporation through patterned masks. Some devices were then annealed through the application of heat (150 °C) for predetermined amount of time. Current-voltage measurements were taken using a Keithley 2400 source meter while the device was under irradiation (100 mW/cm^2^) generated by a Xe arc lamp based Newport 67005 150 W solar stimulator (Franklin, MA, USA) equipped with an AM1.5 filter. The light intensity was calibrated at wavelength 576 nm by a Newport thermopile detector (model 818-010-12) equipped with a Newport 1916-C Optical Power Meter.

### 2.3. Detailed Synthetic Procedures

**Poly(3-hexylthiophene) (P3HT).** One half gram of 2-bromo-3-hexyl-5-iodothiophene (M1, 1.34 mmol), 0.032 g lithium chloride (0.755 mmol) and a stir bar were evacuated under high vacuum overnight in a 100 mL 3 neck round bottom flask. Anhydrous THF (24 mL) was added to the round bottom flask through de-gassed syringe. The solution was cooled down to 0 °C and then 0.67 mL of isopropylmagnesium chloride (2M in THF) was added via a de-gassed syringe. The mixture was allowed to react for ca. 30 min and monitored by NMR. The solution was then warmed to 35 °C and 0.0075 g of dichloro(1,3-bis(diphenylphosphino)propane) nickel (0.0134 mmol), suspended in 2.3 mL of anhydrous THF, was quickly injected into the reaction mixture via a de-gassed syringe. The reaction mixture was stirred for another 10 min before a large excess of methanol was added. The P3HT was recovered by precipitation into methanol and purified by Soxhlet extraction sequentially with methanol, acetone, hexanes, THF and chloroform. The final product was isolated by precipitation of the chloroform solution into methanol and dried at 50 °C under high vacuum for 24 h as a block powder (101.8 mg, 46%). ^1^H-NMR (300.13 MHz, CDCl_3_): δ (ppm) = 6.98 (Th–*H*), 2.80 (Th–C*H*_2_), 1.71 (Th–CH_2_C*H*_2_), 1.40 (Th–CH_2_CH_2_[C*H*_2_]_3_CH_3_), 0.93(Th–CH_2_CH_2_[CH_2_]_3_C*H*_3_). SEC (CHCl_3_, 1 mL/min): *M*_n_ = 19.5 kDa, *M*_w_ = 23.4 kDa, *Đ* = 1.2.

**BCP1.** M1 (1.0 g, 2.68 mmol), 0.576 g lithium chloride (1.34 mmol) and a stir bar were evacuated under high vacuum overnight in a 100 mL 3 neck round bottom flask (RBF1). Simultaneously in a 25 mL two neck flask (RBF2), M2 (0.134 g, 0.268 mmol) was combined with 0.058 g of lithium chloride (0.134 mmol) and a stir bar and were evacuated under high vacuum overnight. Anhydrous THF was added via de-gassed syringes, 50 mL to RBF1 and 5 mL to RBF2, respectfully. Both solutions were cooled down to 0 °C and isopropylmagnesium chloride (2M in THF) was added, 1.98 mL to RBF1 and 1.37 mL to RBF2, via de-gassed syringes. The mixtures were allowed to react for ca. 30 min and monitored by NMR. Both reaction mixtures were warmed to 35 °C and dichloro(1,3-bis(diphenylphosphino)propane) nickel (0.0076 g, 0.0134 mmol) suspended in 2.30 mL of anhydrous THF was injected into the RBF1 via a de-gassed syringe. After 30 min a 0.3 mL aliquot was taken and quenched into an excess of ethylmagnesium bromide and pumped down. SEC (CHCl_3_, 1 mL/min): *M*_n_ = 37.9 kDa, *M*_w_ = 42.7 kDa, *Đ* = 1.1. The contents of RBF2 were transferred into RBF1 via a cannula. The reaction mixture was stirred for another 45 min before 2 mL ethylmagnesium chloride (2M in THF) was added. BCP1 was recovered by precipitation into methanol and purified by Soxhlet extraction sequentially with methanol, acetone, hexanes, THF and chloroform. The final product was isolated by precipitation of the chloroform solution into methanol and dried at 50 °C under high vacuum for 24 h as a block powder (250 mg, 56%). ^1^H-NMR (300.13 MHz, CDCl_3_): δ (ppm) = 6.98 (Th-*H*), 3.65 (C*H*_2_OSiR_3_), 2.80 (Th-C*H*_2_), 1.71–0.83 (alkyl-*H*′s). SEC (CHCl_3_, 1 mL/min): *M*_n_ = 46.6 kDa, *M*_w_ = 52.7 kDa, *Đ* = 1.1.

**BCP2.** In a clean 50 mL single neck round bottom flask, 150 mg of BCP1 was dissolved in 20 mL of anhydrous THF under N2 atmosphere at 60 °C. The solution was stirred at 540 RPM while 0.11 mL of 1 M tetrabutylammonium fluoride (TBAF) solution in THF was added dropwise to the solution. The reaction mixture was stirred for 9 h, then concentrated under reduced pressure and predicated into methanol. BCP2 was further washed with ca. 250 mL of methanol and then dried under high vacuum. (0.129 g, 86%). ^1^H-NMR (300.13 MHz, CDCl_3_): δ (ppm) = 6.98 (Th–*H*), 3.66 (C*H*_2_OH), 2.80 (Th–C*H*_2_), 1.71–0.83 (alkyl–*H*′s). SEC (CHCl_3_, 1 mL/min): *M*_n_ = 32.9 kDa, *M*_w_ = 37.8 kDa, *Đ* = 1.2.

**BCP3.** BCP2 (65.1 mg, 0.0471 mmol –OH groups) and 0.0230 g of 4-dimethylaminopyridine (0.1883 mmol) in 15 mL of anhydrous chlorobenzene were heated to 90 °C under an argon atmosphere until complete dissolution. Nicotinoyl chloride hydrochloride complex (0.0171 g, 0.0961 mmol) was added as a solid and the reaction mixture was stirred for ca. 8 h. BCP3 was recovered by precipitation into methanol and purified by Soxhlet extraction sequentially with methanol, acetone, hexanes, THF and chloroform. The final product was isolated by precipitation of the chloroform solution into methanol and dried at 50 °C under high vacuum for 24 h as a block powder (59.0 mg, 91%). ^1^H-NMR (300.13 MHz, CDCl_3_): δ (ppm) = 9.27, 8.75, 8.28 (Py–*H*′s), 6.98 (Th–*H*), 4.37 (–C*H*_2_OOC–), 2.80 (Th–C*H*_2_), 1.71–0.83 (alkyl–*H*′s). SEC (CHCl_3_, 1 mL/min): *M*_n_ = 44.2 kDa, *M*_w_ = 51.8 kDa, *Đ* = 1.2.

**CdSe Quantum Dots.** CdSe quantum dots were synthesized by using modified procedures from previous reports [44]. A typical synthetic procedure for 3.35 nm CdSe quantum dots was as follows: selenium precursor was prepared by mixing 0.518 g (6.56 mmol) selenium powder and 2 mL tributylphosphine (TBP, 1.62 g, 8.01 mmol) in a scintillation vial for 30 min. Cadmium precursor was prepared by loading 0.042 g CdO (0.33 mmol), 0.386 g stearic acid (SA, 1.36 mmol) 3.88 g hexadecylamine (16.07 mmol) and 3.88 g trioctylphosphine oxide (TOPO, 10.04 mmol) into a 50 mL flask. The initial reddish-brown solution was heated with stirring to 150 °C and kept under N_2_ flow until the color of the solution changed to transparent. Then, the mixture was heated to 320 °C. At this temperature, the Se precursor was quickly injected into the reaction flask. After the injection, the temperature dropped to 290 °C and the reaction was kept for 2 min. After the reaction, the sample was naturally cooled down to room temperature. The product was re-dispersed in chloroform. Then, it was precipitated by acetone followed by centrifugation. The product was washed for three times and dried under vacuum for overnight and re-dispersed in hexane.

**PDTC Ligand.** Concentrated ammonium hydroxide (30 mL, 0.435 mol) and a stir bar were added to a clean 50 mL two necked round bottom flask. While under nitrogen flow and stirring at 1260 RPM, 5 mL of carbon disulfide (0.055 mol) was added dropwise, after which ca. 10 mL of ethanol was added. This resulted in the solution turning an opaque rose color. The solution was immersed into an ice bath and 5 mL of aniline (0.083 mol) was added dropwise to the solution over ca. 5 min. The solution was allowed to react for 45 min when it was removed from the ice bath. The solution was vacuum filtered and the resulting solid was washed with chloroform. Additional solid formed in the filtrate after sometime and the process was repeated until no more solid formed. The pale yellow/white solid was then vacuum dried and stored in the refrigerator (13.7 g, 85%). ^1^H-NMR (300.13 MHz, CDCl_3_): δ (ppm) = 7.49–7.32 (Ph–*H*′s), 7.29–7.26 (–N*H–*).

**PyDTC Ligand.** In a clean 100 mL flask, with a flowing air condenser attached, 30 mL of triethylamine (0.215 mol) and 20.34 g of 2-aminopyridene (0.216 mol) were heated until the solid fully dissolved. While stirring, 14 mL of carbon disulfide (0.238 mol) was added dropwise over 5 min. This solution thickened to form a bright yellow solid over the next 1.5 h, and a mechanical stirrer was applied. The bright yellow solid was recovered by filtration and purified by Soxhlet extraction with diethyl ether and finally dried overnight under high vacuum (11.5 g, 84%). ^1^H-NMR (300.13 MHz, CDCl_3_): δ (ppm) = 9.4 (–N*H*–), 9.0–6.90 (Py–*H*), 3.49–3.23 (NC*H*_2_CH_3_), 1.41–1.36 (NCH_2_C*H_3_*).

**QD Ligand Exchange.** The molarity of CdSe was calculated to be 268,457.59 g/mol [45]. CdSe QD (52 mg, ca. 5 μmol QDs) was added to a clean 20 mL scintillation vial and dissolved in 4 mL of dichloromethane and a few drops of hexane. The solid completely dissolved after 40 min of stirring at 780 RPM. The solution was pipetted into another 20 mL scintillation vial containing 4.59 mg of PDTC (2.68 mmol). The solution was stirred in the dark for the next 82 h. The solution was then concentrated and precipitated into methanol. The quantum dots were then washed several times with hexanes followed by centrifugation and drying under high vacuum (17 mg, 33%).

## 3. Results and Discussion

**Scheme 1:**
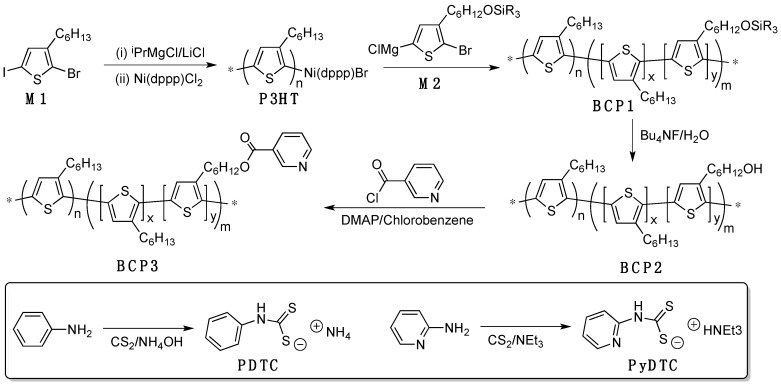
Synthesis of Block Copolymers and Capping Ligands.

**Figure 1:**
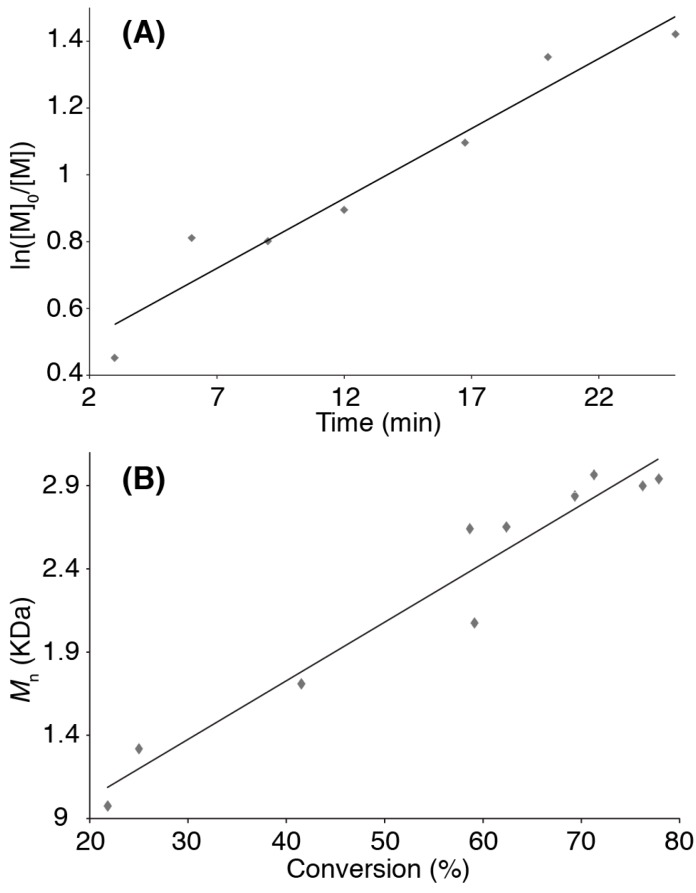
Kinetics plots of GRIM (Grignard metathesis) polymerization of M1: (**A**) ln([M]_0_/[M]) vs. time; and (**B**) *M*_n_ vs. conversion.

Synthetic procedures of the P3HT, functionalized block copolymers (BCPs) and quantum dot (QD) capping ligands are detailed in Scheme 1. The quasi-living Grignard metathesis (GRIM) polymerization techniques were employed [46,47,48]. Both monomers M1 and M2 were synthesized according to literature procedures [27] and sequentially polymerized under conventional GRIM conditions. The kinetics of polymerization of M1 was monitored by NMR spectroscopy and size exclusion chromatography (SEC) and the results are shown in Figure 1. Both the ln([M]_0_/[M]) vs. time (A) and *M*_n_ vs. conversion (B) plots display linear behaviors up to at least 80% monomer conversions, confirming the quasi-livingness of the polymerization techniques employed. M2 was thus added at ca. 80% conversion of M1 and the overall M1/M2 ratio was kept at 10/1. As a result, the second block in BCP1 is expected to be a random copolymer of both monomers and the M1/M2 ratio in the second block is ca. 2/1. From SEC results, the block length ratio between the unfunctionalized P3HT and functionalized block is ca. 4.5/1 with ca. 225 repeat units in the first block and ca. 50 repeat units in the second. Based on the monomer feed ratio and the 80% conversion of M1 at which M2 was added, assuming the polymerization went to close to 100% conversion, there are ca. 17 M2 units on average per polymer chain, leading to the functional group concentration to be ca. 6% that matches closely with the 7% functional group concentration calculated from ^1^H-NMR integration.

**Figure 2:**
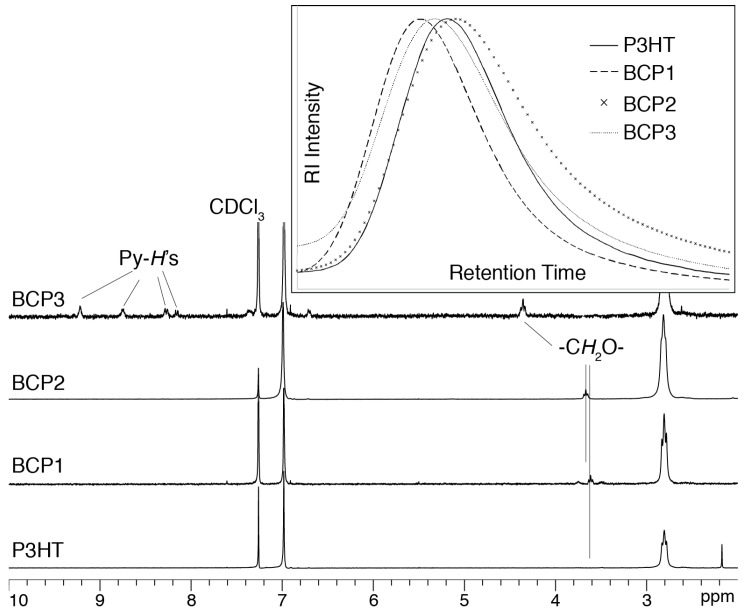
^1^H-NMR overlay of P3HT, BCP1, BCP2 and BCP3 in selected regions. Insert: size exclusion chromatograms (SEC) of the same polymers (RI, CHCl_3_, 1 mL/min).

The *tert*-butyldimethylsilyl protecting groups in BCP1 were removed by using tetrabutylammonium fluoride in THF, leading to the hydroxy functionalized BCP2. Reaction of BCP2 with an excess of nicotinoyl chloride in the presence of a base smoothly converted the hydroxy groups to pyridine (Py) functionalities. The reaction sequence was monitored by ^1^H-NMR spectroscopy as shown in Figure 2. The chemical shift regions excluding most of the aliphatic protons were selected for clarity. In the spectrum of P3HT, only one singlet at 6.98 ppm and a broad triplet at 2.80 ppm were present, corresponding to the aromatic Th–*H* and methylene group directly attached to the thiophene rings (Th–C*H*_2_), respectively. These two signals are expectedly present in the spectra of all other polymers. Upon chain extension, a new triplet at ca. 3.65 ppm appears in the spectrum of BCP1, corresponding to the methylene protons attached to the silylether groups. Size exclusion chromatography (SEC) traces of both P3HT and BCP1 (Figure 2, insert) display well-defined and narrow peaks corresponding to Mn values of 37.9 and 46.6 kDa, respectively. The polydispersity (*Đ*) indices of both polymers are ca. 1.1, and such narrow distributions and the lack of P3HT signals in the SEC trace of BCP1 suggest quantitative chain extension and formation of well-defined block copolymers. Upon removal of the silyl protecting groups, BCP2 shows very similar NMR spectrum as that of BCP1, except for the hydroxy methylene proton signals shift slightly to 3.66 ppm. However, the SEC trace of BCP2 shifts to longer elution time with apparent tailing effects. We suspect that interactions of the polar hydroxy groups in BCP2 with SEC column materials are responsible for the seemingly decreased molecular weight and broadened polydispersity. These hydroxy groups were then smoothly converted into pyridine functionalities through esterification reactions with nicotinoyl chloride, leading to the final product BCP3. The signals from the oxygen bound methylene protons completely shift to 4.37 ppm together with the appearance of pyridine proton signals between 8 and 9.2 ppm. Integration of these signals also give ca. 7% functionalization percentage, confirming the quantitative transformation during the series of reactions. The SEC trace of BCP3 shifts to a smaller retention time than that of BCP2 but the molecular weight is still slightly smaller than expected, presumably caused by interactions between the pyridine groups and column packing materials.

**Figure 3:**
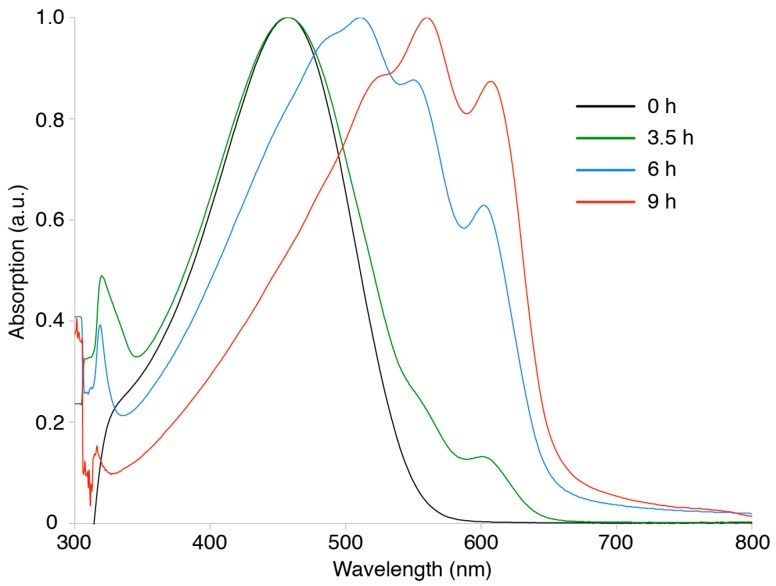
UV-vis absorption spectra of BCP3 in chlorobenzene/acetone (4/1, *v*/*v*) at various time delays.

**Figure 4:**
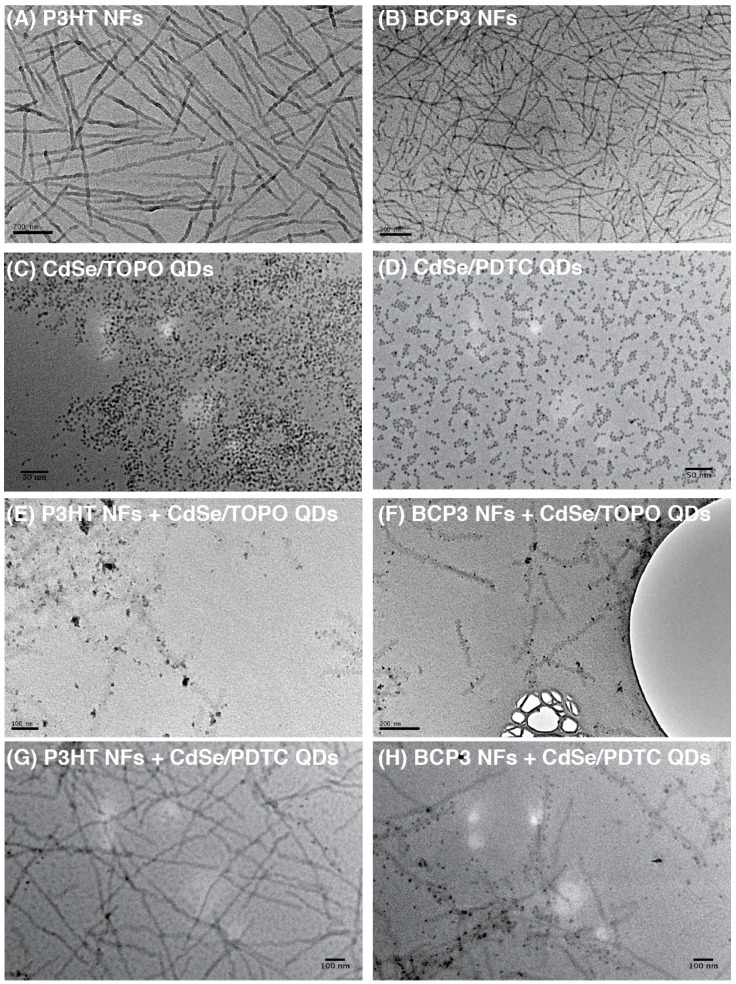
Transmission electron microscopy (TEM) images of: (**A**) P3HT nanofibers (NF)s; (**B**) BCP3 NFs; (**C**) CdSe quantum dots (QDs) with TOPO ligands; (**D**) CdSe QDs with PTDC ligands; (**E**) P3HT NFs with CdSe QDs having TOPO ligands (1/1, *w*/*w*); (**F**) BCP3 NFs with CdSe QDs having TOPO ligands (1/1, *w*/*w*); (**G**) P3HT NFs with CdSe QDs having PTDC ligands (1/1, *w*/*w*); and (**H**) BCP3 NFs with CdSe QDs having PTDC ligands (1/1, *w*/*w*).

We then prepared nanofibers (NFs) from both P3HT and BCP3 by using the mixed solvent approach. Briefly, 5 mg polymer was dissolved in 1 mL chlorobenzene, a good solvent for both P3HT and the pyridine functionalized block. Then 0.25 mL acetone was added and the mixture was stirred slowly at room temperature. Aliquots were taken at pre-determined time interval, diluted 100 times with chlorobenzene/acetone (4/1, *v*/*v*) and subjected to UV-vis absorption measurements. Due to the high polarity, acetone is a poor solvent for the P3HT block, while being a good solvent for pyridine functionalities. Addition of acetone is thus expected to induce crystallization of the pure P3HT block into NFs while solvate the pyridine functionalized block as a corona outside the NF core. As shown in Figure 3, the UV-vis spectrum of BCP3 in the mixture solvent right after acetone addition displays a structureless profile having λ_max_ at ca. 454 nm, indicating that the polymer is well dissolved at this stage. With time, the absorption spectra gradually red-shift and become structured. At 9 h, the three vibronic features at ca. 518, 555 and 605 nm became apparent, after which the spectrum did not change significantly. We thus prepared all polymer NFs by aging the mixed solvent solutions for 9 h. Transmission electron microscopy (TEM) measurements confirmed the formation of NFs as shown in Figure 4A,B. P3HT homopolymers formed uniform NFs having an average width of ca. 15 nm and lengths up to microns. On the other hand, BCP3 also form NFs with an average width of ca. 15 nm while the dispersity of fiber length is quite large. We suspect that the pyridine groups in BCP3 may induce additional interactions among the polymer themselves and interfere with the NF formation by the P3HT block.

CdSe quantum dots (QDs) were prepared according to slightly modified literature procedures [44] and the as-prepared QDs have an average diameter of 3.33 ± 0.31 nm by TEM analysis and trioctylphosphine oxide (TOPO) as the ligand shell. These QDs have limited solubility in chlorobenzene and acetone used for NF formation, but are very soluble in dodecane. UV-vis absorption profiles of the QDs give a λ_max_ at ca. 596 nm, which led to a calculated QD size of ca. 4.4 nm [49]. TEM image of these QDs are displayed in Figure 4C, in which the QDs are uniform in sizes and more or less dispersed without significant aggregation. To study the self-assembly processes of polymer NFs with CdSe QDs, we added equal weight of QDs in minimum amount of dodecane into the pre-formed NF solutions. The same amount of pure dodecane did not cause any appearance changes or absorption changes of the NF solutions, confirming that dodecane did not change the NFs alone. The resulting NF/QD solutions were diluted and cast onto carbon-coated TEM grids and the images are shown in Figure 4E,F. In the case of P3HT NFs, the CdSe QDs are found to preferentially located in areas where the NFs are present. However, most of the QDs are not closely associated with the NFs. This phenomenon is understandable since the side-chains in P3HT and TOPO ligands are both alkyl chains so that weak hydrophobic interactions bring these two components near each other. On the other hand, there are no specific interactions between these two compounds so that they are not strongly associated with one another. Things are quite different when BCP3 NFs are applied since the pyridine functionalities should have stronger coordinating interactions with the inorganic QDs and we expected to observe closer interactions between the NFs and QDs. Indeed, as seen in Figure 4F, the CdSe QDs are also concentrated in areas where the NFs are present and most of the QDs are attached to the peripheries of the NFs. Such core/shell organic/inorganic composite NF structures provide a facile means to control the nanostructures and morphologies of hybrid materials.

The currently applied TOPO ligands form a thick, non-conductive layer outside the QDs, potentially limiting electronic communications between the organic CPs and QDs. We thus replaced these alkyl ligands with shorter phenyldithiocarbamate (PDTC) and pyridyldithiocarbamate (PyDTC) ones as shown in Scheme 1. Both ligands led to similar QD behavior so far and we will focus our attention on the PDTC ligands in the current studies. The TEM image in Figure 4D shows the QDs with PDTC ligands, from which an average diameter of ca. 3.03 ± 0.15 nm. Such size reduction is expected from the shorter PDTC ligands. UV-vis absorption measurements gave a red-shift of λ_max_ to ca. 605 nm, which has been previously attributed to QD to ligand charge transfer interactions [50]. The TEM image shows clusters of QDs and such aggregation effects are possibly caused by stronger interactions among the rigid phenyl groups in PDTC ligands. The PDTC coated CdSe QDs are soluble in chlorobenzene but only poorly dissolved in the chlorobenzene/acetone mixture used for NF formation. For self-assembly studies, an alternative route was taken by dissolving the polymers and QD (1/1, *w*/*w*) in chlorobenzene first and then adding acetone. The process was monitored by using UV-vis absorption spectroscopy and showed very similar behaviors compared with those without QDs. TEM images of the resulting composite solutions are shown in Figure 4G,H. Both P3HT and BCP3 form similar NFs as those prepared in the absence of QDs. Very few QDs were found where most P3HT NFs reside (Figure 4G), indicating no specific interactions between these two components. On the other hand, in Figure 4H, large quantities of QDs are clearly found near the BCP3 NFs and seemingly line up along both sides of the NF with similar distances. Such behaviors can only be explained by the non-covalent interactions between BCP3 and QDs bearing PTDC ligands. We also performed fluorescence quenching experiments on the NFs of both BCP3 and P3HT by using QDs bearing PDTC ligands and found that the quenching constant of BCP3 NFs is circa five times larger than that of P3HT NFs (Figure S1, Supporting Information), further confirming the stronger association between BCP3 NF and CdSe QDs.

Organic solar cell devices were fabricated using the conventional device structure: ITO glass/MoO_3_ (10 nm)/active layer (100 nm)/Al (80 nm). The active layers contain polymers, either P3HT or BCP3, CdSe QDs having PDTC ligands, and PCBM at a constant 1:1:1 weight ratio for better comparison. All devices were thermally annealed at 150 °C for 10 min under N_2_ and the results are summarized in Table 1. BHJ devices are simple blends of all components from a common solution in chlorobenzene, in contrast to the NF devices, in which polymer NFs were formed first in chlorobenzene/acetone mixtures before QDs and PCBM were added. As summarized in Table 1, addition of QDs significantly decrease device performances when compared with binary devices of P3HT/PCBM we reported recently [30]. The devices suffer greatly from both open circuit voltage (*V*_OC_) and fill factors (*FF*) values, indicating severe energy loss during charge separation and transport processes. Weiss et al. have recently studied the ligand shell effects on electronic properties of QDs and found that PDTC ligands act as hole acceptors when combined with CdSe QDs [51]. Based on such energy landscape, these QDs may in fact act as recombination centers and significantly reduce obtained voltages while decrease diode ideality. Although the BCP3 devices performed slightly better than P3HT devices, which is likely due to better morphologies from the self-assembly behaviors, QDs with a different ligand sets that have the correct energy alignment with both P3HT and PCBM are needed to truly investigate the effectiveness of the ternary core–shell NF structures on device performance.

## 4. Conclusions

In summary, we have prepared a novel conjugated block copolymer, BCP3, based on P3HT backbone having selectively functionalized pyridine moieties, which forms well-defined nanofibers (NFs) in mixture solvents. Self-assembly of such BCP3 NFs with CdSe quantum dots (QDs) in solutions led to the formation of core–shell organic/inorganic composite NFs. Such strategy provides a unique opportunity to control the special arrangement between incompatible components and potential benefits in organic electronic devices including photovoltaics. However, the PDTC ligand shells were found to act adversely toward OSC performances in ternary polymer/QD/PCBM devices and we are currently investigating other possible ligand sets for QDs that have the correct electronic requirements to allow desired charge transfer processes.

## Figures and Tables

**Table 1:** Summary of Solar Cell Device Performance Data ^a^.

Blends ^b^	*PCE* (%) ^c^	*J*_SC_ (mA/cm^2^) ^d^	*V*_OC_ (V) ^e^	*FF* (%) ^f^
P3HT BHJ	0.17 ± 0.03 (0.19)	1.89 ± 0.156 (2.00)	0.27 ± 0.00 (0.27)	33 ± 3 (31)
P3HT NF	0.53 ± 0.21 (0.79)	3.95 ± 1.54 (5.99)	0.51 ± 0.02 (0.54)	27 ± 2 (28)
BCP3 BHJ	0.78 ± 0.08 (0.87)	4.23 ± 0.618 (4.92)	0.49 ± 0.01 (0.50)	38 ± 1 (40)
BCP3 NF	0.42 ± 0.06 (0.46)	3.26 ± 1.15 (4.35)	0.34 ± 0.04 (0.39)	41 ± 14 (58)

^a^ All numbers are reported as averages from at least five devices, highest values are included in parentheses; ^b^ All blends have polymer/QD/PCBM weight ratio at 1/1/1 and thermally annealed at 150 °C for 10 min under N_2_; ^c^ Power conversion efficiency; ^d^ Short circuit current density; ^e^ Open circuit voltage; ^f^ Fill factor.

## Supplementary Materials

The following are available online at www.mdpi.com/2073-4360/8/12/408/s1, Figure S1: Fluorescence spectra of P3HT NFs (**A**) and BCP3 NFs (**B**) in the presence of QDs with PDTC ligands of various weight ratios. Inserts: corresponding Stern-Volmer plots.
