# Supplementary material for: Core/Shell Conjugated Polymer/Quantum Dot Composite Nanofibers through Orthogonal Non-Covalent Interactions
Source: Polymers (Basel). 2016 Nov 24;8(12):408. doi: 10.3390/polym8120408 (PMC6432181; doi:10.3390/polym8120408)
Supplement: Supplementary file 1 [file polymers-08-00408-s001.pdf]

Supplementary Materials: Core/Shell Conjugated Polymer/Quantum Dot Composite Nanofibers through Orthogonal Non-Covalent Interactions

Brad W. Watson II, Lingyao Meng, Chris Fetrow and Yang Qin

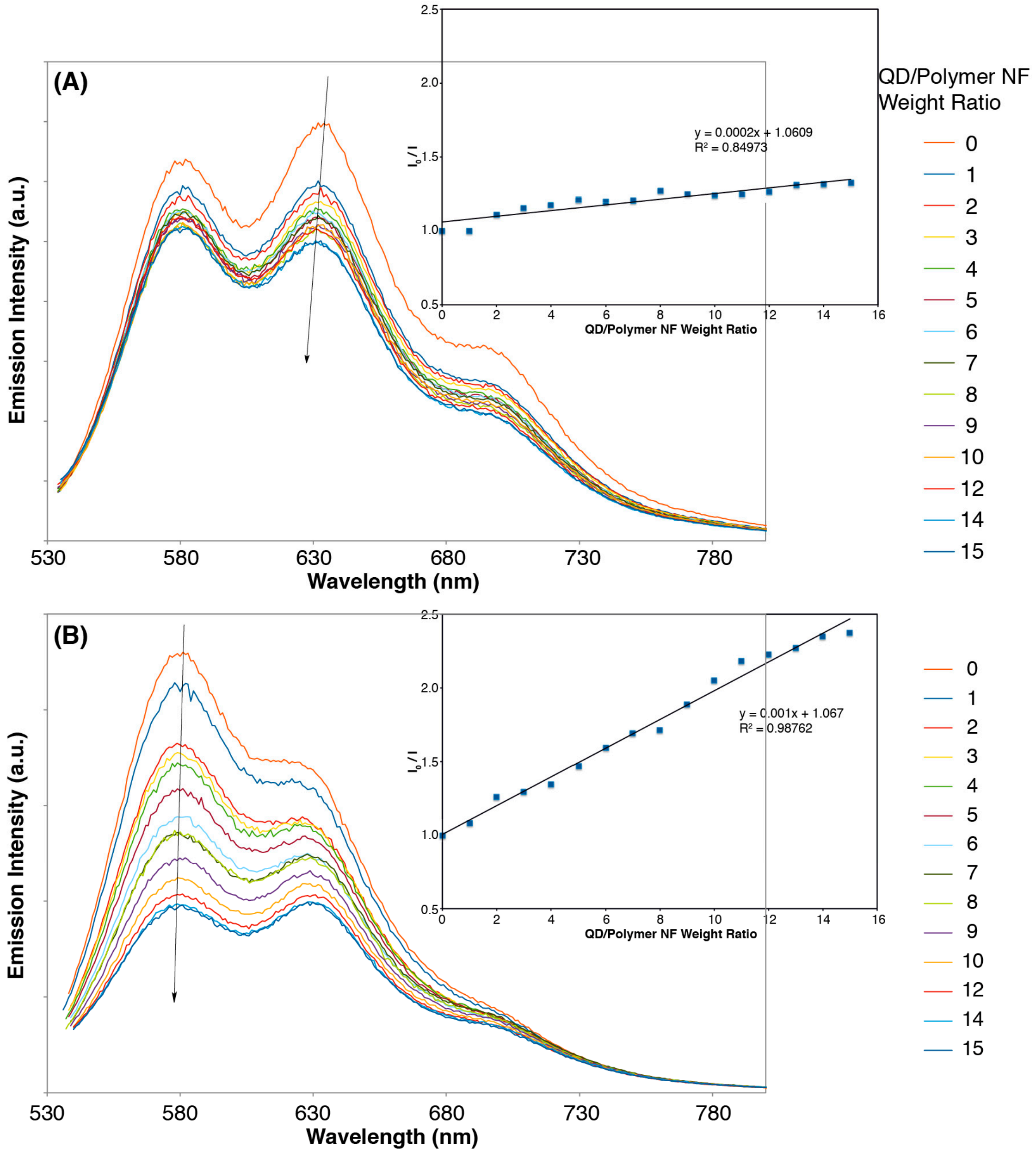

Figure S1. Fluorescence spectra of P3HT NFs (**A**) and BCP3 NFs (**B**) in the presence of QDs with PDTC ligands of various weight ratios. Inserts: corresponding Stern-Volmer plots.
